# Clinicopathological parameters, recurrence, locoregional and distant metastasis in 115 T1-T2 oral squamous cell carcinoma patients

**DOI:** 10.1186/1758-3284-2-9

**Published:** 2010-04-20

**Authors:** Waseem Jerjes, Tahwinder Upile, Aviva Petrie, Andrew Riskalla, Zaid Hamdoon, Michael Vourvachis, Kostas Karavidas, Amrita Jay, Ann Sandison, Gareth J Thomas, Nicholas Kalavrezos, Colin Hopper

**Affiliations:** 1 UCLH Head and Neck Centre, London, UK; 2 Department of Surgery, University College London Medical School, London, UK; 3 Unit of Oral & Maxillofacial Surgery, Division of Maxillofacial, Diagnostic, Medical and Surgical Sciences, UCL Eastman Dental Institute, London, UK; 4 Biostatistics Unit, UCL Eastman Dental Institute, London, UK; 5 Department of Pathology, University College London, London, UK; 6 Department of Histopathology, Imperial College and Hammersmith Hospitals, London, UK; 7 Department of Pathology, Southampton University School of Medicine, Cancer Sciences Division, Southampton, UK

## Abstract

The incidence of oral squamous cell carcinoma remains high. Oral and oro-pharyngeal carcinomas are the sixth most common cancer in the world. Several clinicopathological parameters have been implicated in prognosis, recurrence and survival, following oral squamous cell carcinoma. In this retrospective analysis, clinicopathological parameters of 115 T1/T2 OSCC were studied and compared to recurrence and death from tumour-related causes.

The study protocol was approved by the Joint UCL/UCLH committees of the ethics for human research. The patients' data was entered onto proformas, which were validated and checked by interval sampling. The fields included a range of clinical, operative and histopathological variables related to the status of the surgical margins. Data collection also included recurrence, cause of death, date of death and last clinic review. Causes of death were collated in 4 categories (1) death from locoregional spread, (2) death from distant metastasis, (3) death from bronchopulmonary pneumonia, and (4) death from any non-tumour event that lead to cardiorespiratory failure.

The patients' population comprised 65 males and 50 females. Their mean age at the 1^st ^diagnosis of OSCC was 61.7 years. Two-thirds of the patients were Caucasians. Primary sites were mainly identified in the tongue, floor of mouth (FOM), buccal mucosa and alveolus. Most of the identified OSCCs were low-risk (T1N0 and T2N0). All patients underwent primary resection ± neck dissection and reconstruction when necessary. Twenty-two patients needed adjuvant radiotherapy. Pathological analysis revealed that half of the patients had moderately differentiated OSCC. pTNM slightly differed from the cTNM and showed that 70.4% of the patients had low-risk OSCC. Tumour clearance was ultimately achieved in 107 patients. Follow-up resulted in a 3-year survival of 74.8% and a 5-year survival of 72.2%.

Recurrence was identified in 23 males and 20 females. The mean age of 1^st ^diagnosis of the recurrence group was 59.53 years. Most common oral sites included the lateral border of tongue and floor of mouth. Recurrence was associated with clinical N-stage disease. The surgical margins in this group was evaluated and found that 17 had non-cohesive invasion, 30 had dysplasia at margin, 21 had vascular invasion, 9 had nerve invasion and 3 had bony invasion. Severe dysplasia was present in 37 patients. Tumour clearance was achieved in only 8 patients. The mean depth of tumour invasion in the recurrence group was 7.6 mm.

An interesting finding was that 5/11 patients who died of distant metastasis had their primary disease in the tongue. Nodal disease comparison showed that 8/10 patients who died of locoregional metastasis and 8/11 patients who died from distant metastasis had clinical nodal involvement. Comparing this to pathological nodal disease (pTNM) showed that 10/10 patients and 10/11 patients who died from locoregional and distant metastasis, respectively, had nodal disease. All patients who died from locoregional and distant metastasis were shown to have recurrence after the primary tumour resection.

Squamous cell carcinoma of the oral cavity has a poor overall prognosis with a high tendency to recur at the primary site and extend to involve the cervical lymph nodes. Several clinicopathological parameters can be employed to assess outcome, recurrence and overall survival.

## Background

The incidence of oral squamous cell carcinoma (OSCC) remains high [1]. Oral and oro-pharyngeal carcinomas are the sixth most common cancer in the world [2]. Despite evolution in management, the overall survival of patients has not improved significantly during the past 20 years, with 5-year survival rates between 45-50% [1].

Several clinicopathological parameters have been implicated in prognosis, recurrence and survival, following oral squamous cell carcinoma. The overall national 5-year survival has been reported to vary in range according to tumour size (T1/T2 commonly referred to as "low-risk tumours" and T3/T4 commonly referred to as "high-risk"). The outcome is greatly influenced by the stage of the disease (especially _pathological_TNM) [3].

Prognosis also depends or varies with tumour primary site, nodal involvement, tumour thickness, and the status of the surgical margins. Moreover, the cumulative effects of tobacco, betel nut and alcohol decrease the survival rate [4].

In this retrospective analysis, the clinicopathological parameters of 115 T1/T2 OSCC patients were studied and correlated to recurrence and death from tumour-related causes.

## Methods

**Figure 1:**
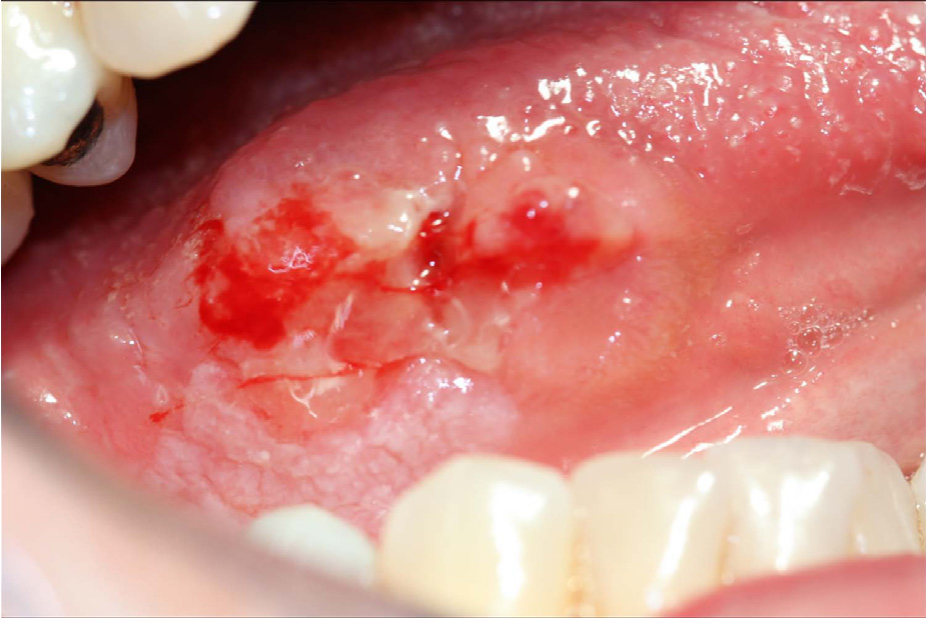
**T1/T2 SCC of the lateral tongue**.

Identical 'intent to treat' protocols were used to treat 115 consecutive patients who presented with T1/T2 oral squamous cell carcinoma (OSCC), (Figure 1) to the Department of Oral and Maxillofacial Surgery, Eastman Dental and University College Hospitals between 1992 and 2001. The study protocol was approved by the Joint UCL/UCLH committees of the ethics for human research.

All patients were operated upon with the primary objective of achieving a macroscopic clearance of 0.5-1.0 cm. Postoperative radiotherapy was given according to our standard protocols, if applicable.

**Figure 2:**
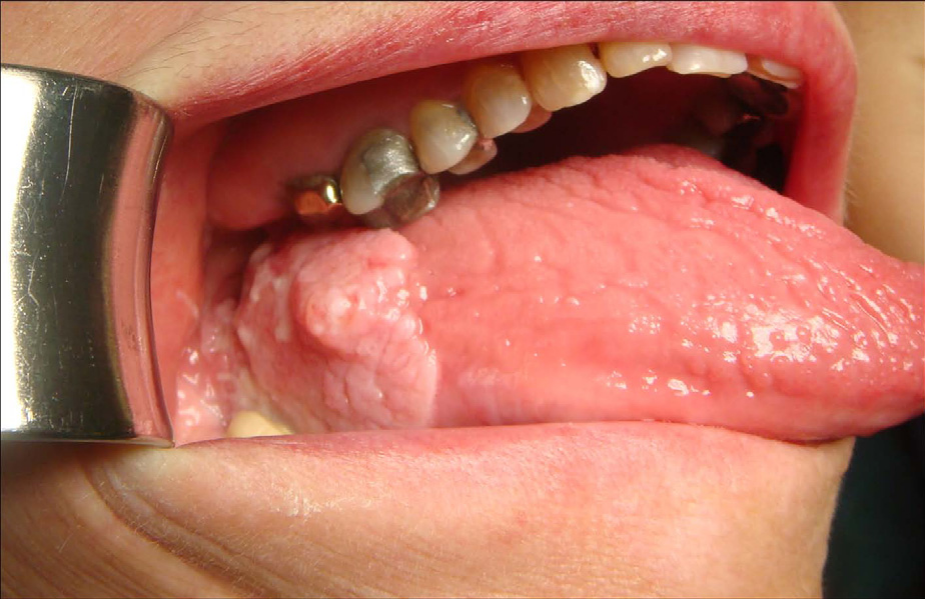
**Recurrence and locoregional spread-SCC of the lateral tongue, floor of mouth, retromolar trigone with extension to the lateral pharyngeal wall**.

**Figure 3:**
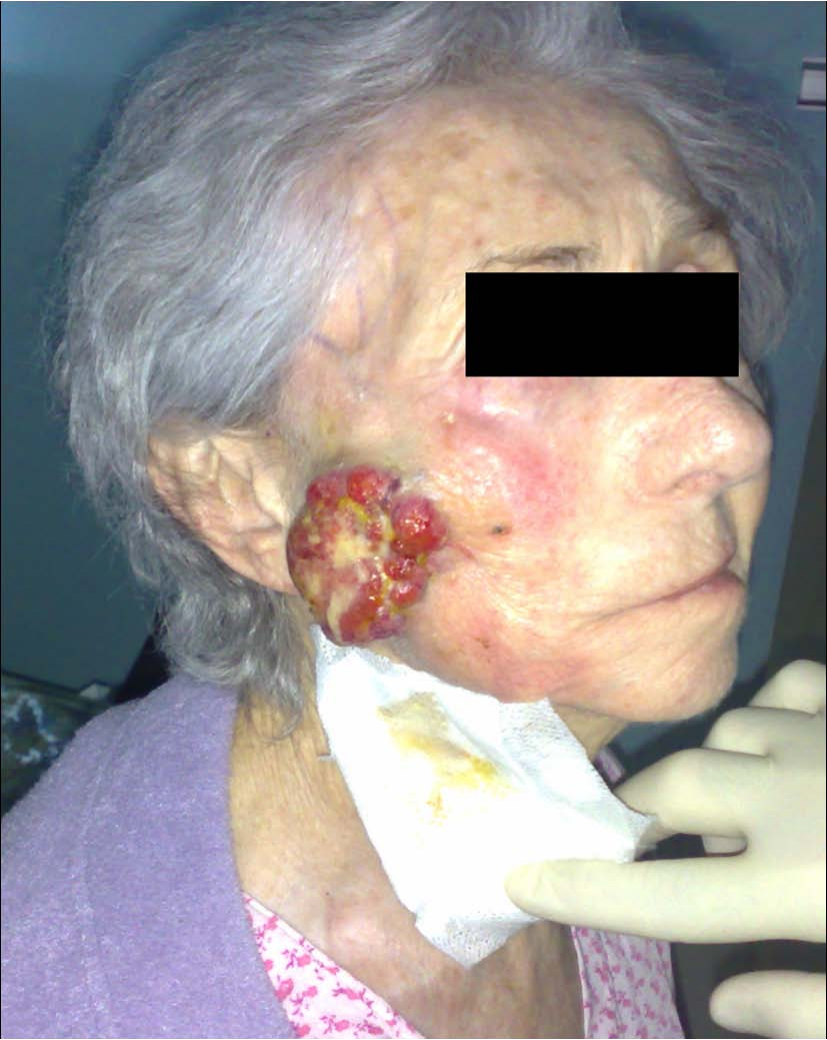
**Recurrence and locoregional spread-exophytic SCC of the right face directly extended from the oropharyngeal region**.

**Figure 4:**
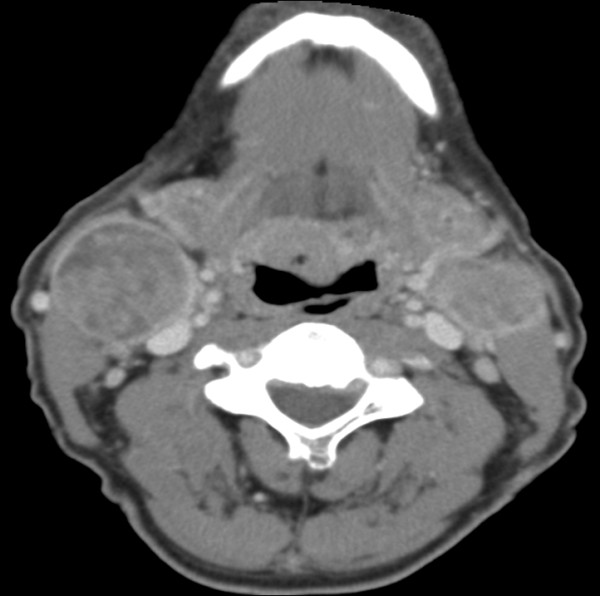
**Recurrence and locoregional spread-bilateral cervical lymphadenopathy of an oral cancer patient**.

**Figure 5:**
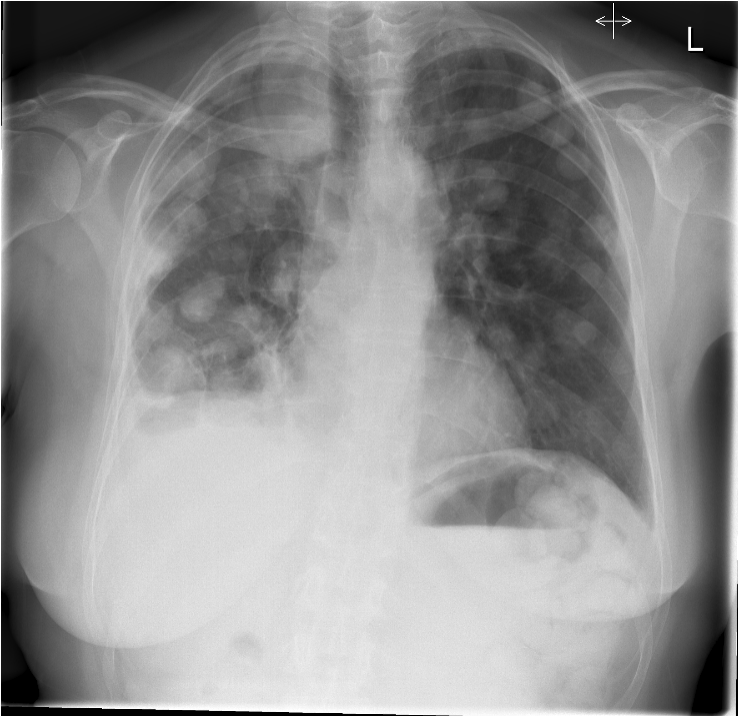
**Distant metastasis-PA chest X-ray showing extensive cannon ball metastasis of the lungs**.

**Figure 6:**
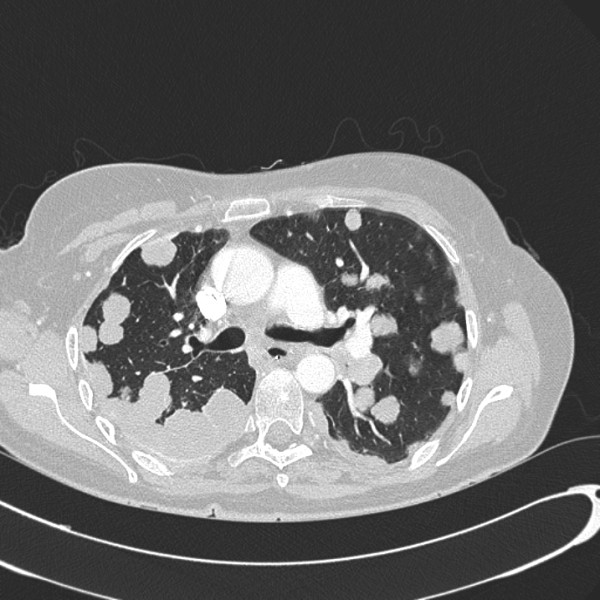
**Distant metastasis-axial chest CT showing tumour metastasis to the plural spaces and parenchyma of the lungs**.

**Figure 7:**
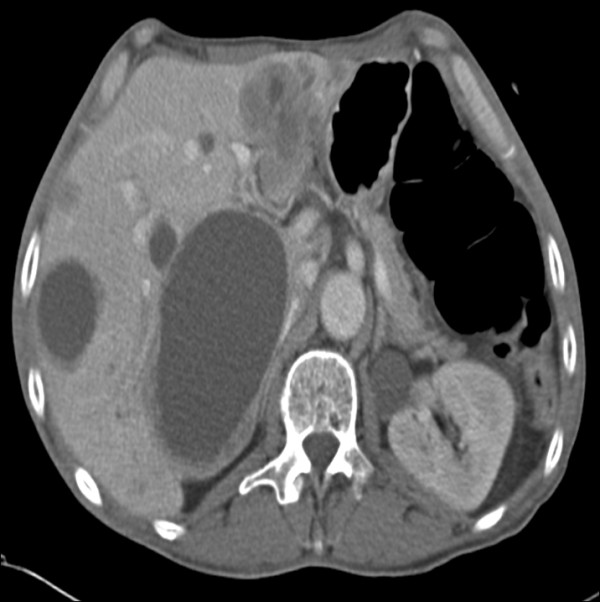
**Distant metastasis-axial upper abdominal CT showing multiple tumour deposits and cysts in the liver**.

The patients' data was entered onto proformas, which were validated and checked by interval sampling. The fields included a range of clinical, operative and histopathological variables related to the status of the surgical margins. Data collected also included recurrence, cause of death, date of death and last clinic review. Causes of death were collated in 4 categories (1) death from locoregional spread (Figures 2, 3 and 4), (2) death from distant metastasis (Figures 5, 6 and 7), (3) death from bronchopulmonary pneumonia, and (4) death from any non-tumour event that lead to cardiorespiratory failure.

### Statistical analysis

The outcomes of the categorical clinicopathological variables were summarised as frequencies and percentages for the whole group of patients and for the subgroups categorised by recurrence, 3 and 5 years survival and cause of death. The numerical variables, "age at 1^st ^diagnosis of SCC" and "depth of invasion (mm)", were summarised by means, standard deviations, minimal and maximal values.

Two way contingency tables were created to investigate the relationship between the categories of the categorical clinicopathological variables and both recurrence and cause of death, and Fisher's exact tests were used to test for statistical significance of the findings. Because the expected number of patients within sub-groups was small, the Kruskal-Wallis test was used to determine if there was a statistically significant difference in the distribution of the numerical variables, the "age at 1^st ^diagnosis of SCC" and "depth of invasion (mm)", for the different categories of recurrence and cause of death.

Logistic regression, using death at the outcome of interest separately for 3-year and 5-year survival was performed to assess the independent effect of the numerical and categorical covariates on the relevant outcome. A Cox proportional hazards survival analysis was performed to assess the independent effect of each of the covariates on survival time, measured in months. A 5% significance level was used to assess the significance of the hypothesis tests and the covariates in the logistic and Cox analyses.

## Results

The patient population comprised 65 males (56.5%) and 50 females (43.5%). Their mean age at the 1^st ^diagnosis of OSCC was 61.7 (SD5.8 years, Min 20 years, Max 96 years). Two-thirds of the patients were Caucasians (67.8%); other prominent racial groups included Africans (11.3%), Indians (8.7%) and Caribbeans (4.3%), (Table 1).

**Table 1:** Demographic details of 115 patients with oral squamous cell carcinoma

	Frequency (%)		Frequency (%)
**Gender**		**Differentiation**	
Male	65 (56.5)	Well	32 (27.8)
Female	50 (43.5)	Moderate	60 (52.2)
		Moderate-poorly	11 (9.6)
**Race**		Poorly	12 (10.4)
Caucasian	78 (67.8)		
Indian	10 (8.7)	**pTNM**	
Middle-Eastern	2 (1.7)	T1N0M0	58 (50.4)
Oriental	1(0.9)	T2N0M0	23 (20.0)
Other Asians	6 (5.2)	T1N1M0	6 (5.2)
African	13 (11.3)	T2N1M0	6 (5.2)
Caribbean	5 (4.3)	T1N2aM0	6 (5.2)
		T2N2aM0	9 (7.8)
**Primary site**		T1N2bM0	1 (0.9)
Floor of mouth	24 (20.9)	T2N2bM0	3 (2.6)
Tongue (lateral)	36 (31.3)	T1N2cM0	2 (1.7)
Tongue (dorsal)	13 (11.3)	T2N2cM0	1 (0.9)
Tongue (ventral)	5 (4.3)		
Buccal mucosa	11 (9.6)	**Invasive front (IF)**	
Hard palate	3 (2.6)	Cohesive	82 (71.3)
Upper alveolus	6 (5.2)	Non-cohesive	33 (28.7)
Lower alveolus	6 (5.2)	**Dys. At Margin**	53 (46.1)
Retromolar area	3 (2.6)	**Lymphvascular Invasion**	28 (24.3)
Tuberosity	1 (0.9)	**Nerve Invasion**	12 (10.4)
Upper lip	1 (0.9)	**B/C Invasion**	5 (4.3)
Lower lip	5 (4.3)	**SD present**	72 (62.6)
Neck Lump*	1 (0.9)	**Tumour clearance**	107 (93.0)
			
**cTNM**		**Recurrence**	43 (37.4)
T1N0M0	62 (53.9)		
T2N0M0	24 (20.9)	**Recurrence Rx**	
T1N1M0	3 (2.6)	Surgery	2 (1.7)
T2N1M0	5 (4.3)	Surgery + radio	13 (11.3)
T1N2aM0	5 (4.3)	Radio + chemo	5 (4.3)
T2N2aM0	9 (7.8)	PDT	2 (1.7)
T1N2bM0	1 (0.9)	Radiotherapy	21 (18.3)
T2N2bM0	3 (2.6)		
T1N2cM0	3 (2.6)	**3 year survival**	86 (74.8)
			
**Primary Rx**		**5 year survival**	83 (72.2)
Surgery	90 (78.3)		
Surgery + radio	22 (19.1)		
Surgery + radio + chemo	3 (2.6)		

**Age at 1st OSCC**		**Depth of Invasion (mm)**	
Minimum	20	Minimum	1.0
Maximum	96	Maximum	18.0
Mean	61.70	Mean	5.657

*Primary site was identified before surgery and staged by cTNM, hence no T0 in the table.

Primary sites were mainly identified in the tongue (46.9%), floor of mouth (FOM) (20.9%), buccal mucosa (9.6%) and alveolus (10.4%). Most of the identified OSCCs were low-risk (T1N0 and T2N0) (74.8%); while the rest had nodal disease, but no distant metastasis was reported. All patients underwent primary resection ± neck dissection and reconstruction when necessary. Twenty-two patients needed adjuvant radiotherapy and 3 others adjuvant chemoradiotherapy (Table 1).

**Figure 8:**
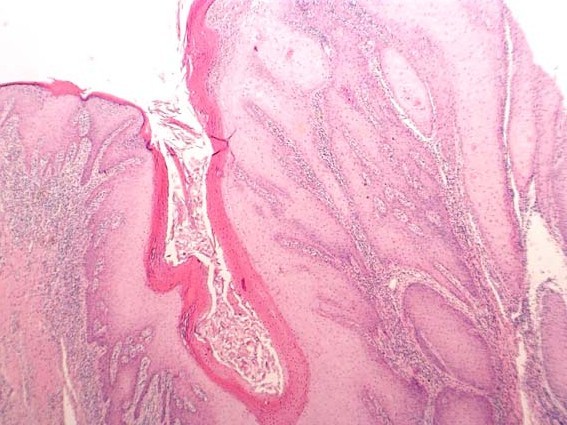
**SCC grading-HE stained section low power ×25 showing well differentiated squamous cell carcinoma (verrucous type)associated with surface hyperkeratosis and inflammation at the epithelial stromal interface**.

**Figure 9:**
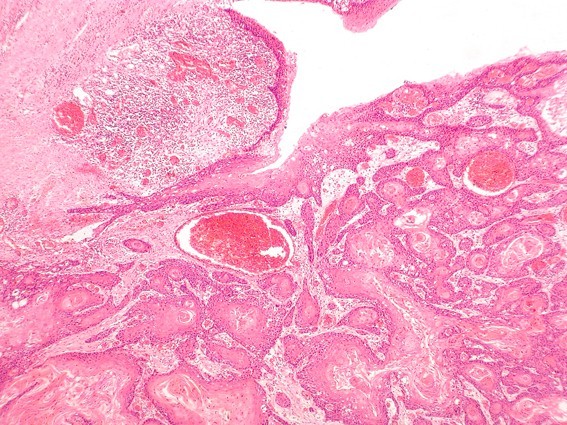
**SCC grading-HE stained section viewed at low power ×25 showing moderately differentiated squamous cellcarcinoma arising from surface epithelium**.

**Figure 10:**
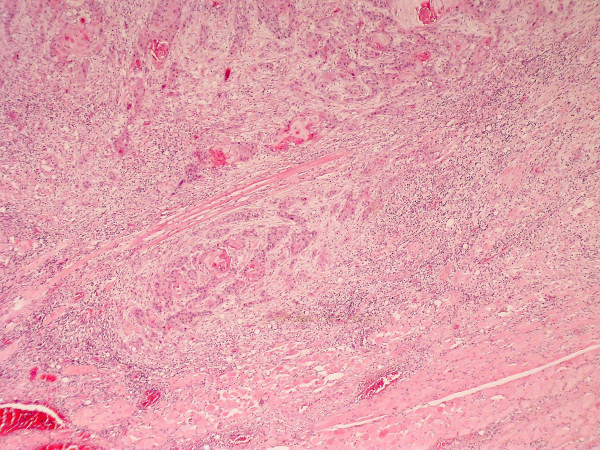
**SCC grading-HE stained sections viewed at low power ×25 showing poorly differentiated squamous cell carcinoma infiltrating as poorly cohesive single cells and nests of tumour cells**. There is no clear demarcation between the tumour invasion front and surrounding tissue.

**Figure 11:**
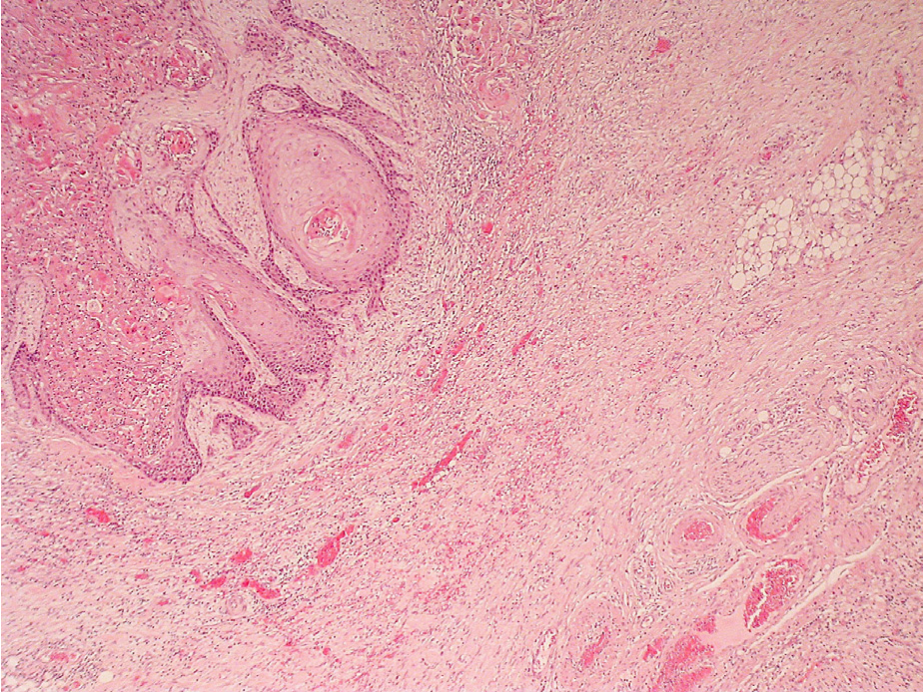
**Pattern of invasion**. HE stained section viewed at low power showing moderately differentiated squamous cell carcinoma with cohesive invasion front. There is a clear demarcation between tumour and surrounding connective tissue.

**Figure 12:**
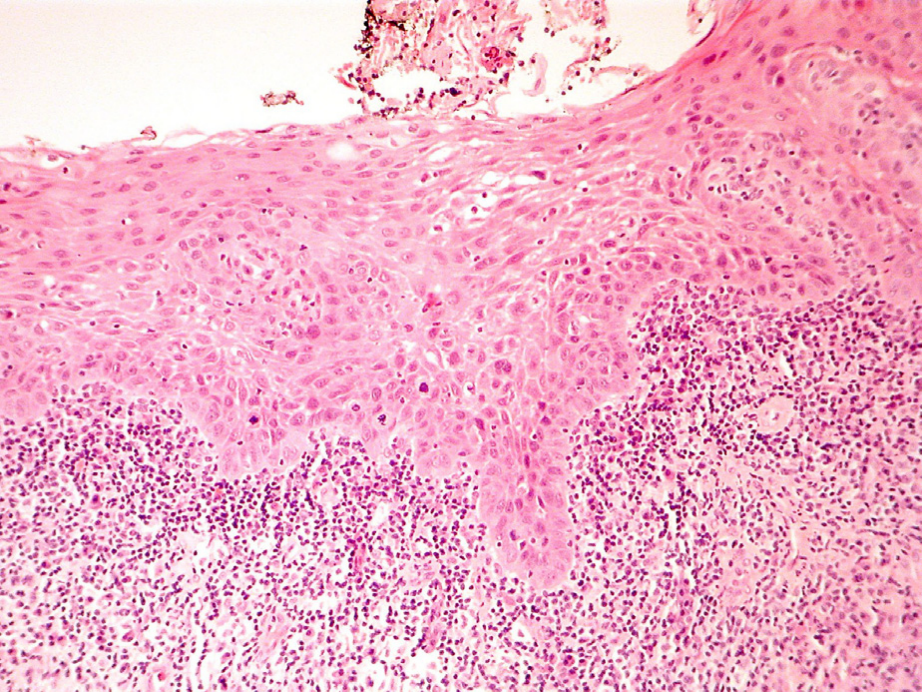
**HE stained section ×50 magnification showing severe dysplasia of surface epithelium**. There is an associated chronic inflammatory infiltrate at the interface between stroma and dysplastic epithelium.

**Figure 13:**
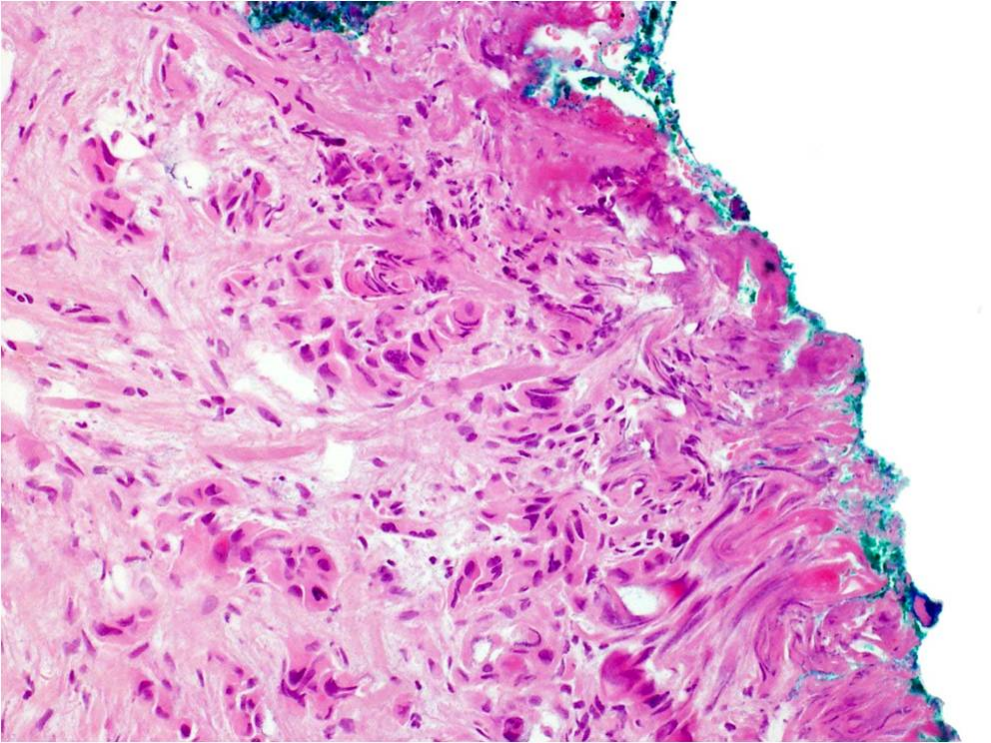
**Tumour depth-HE stained section ×100 magnification showing SCC at submucosal margin**.

**Figure 14:**
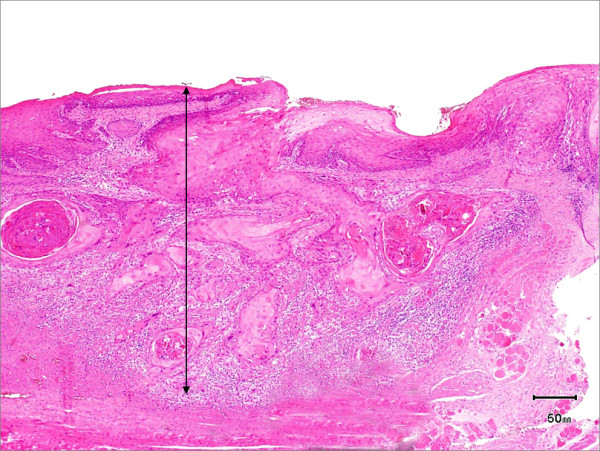
**Tumour depth-HE stained section ×50 magnification showing depth of invasion**.

**Figure 15:**
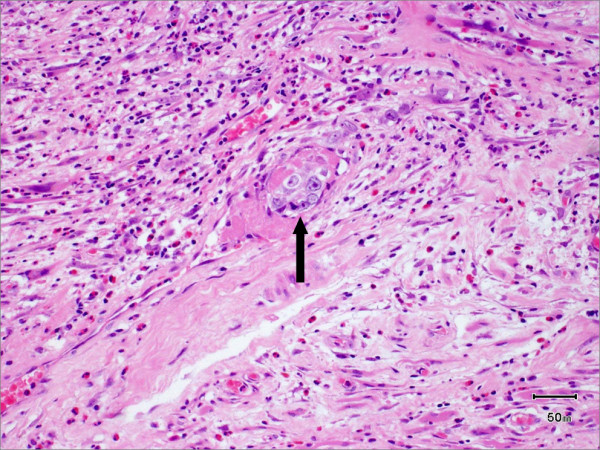
**HE stained section ×100 magnification showing vascular invasion**.

**Figure 16:**
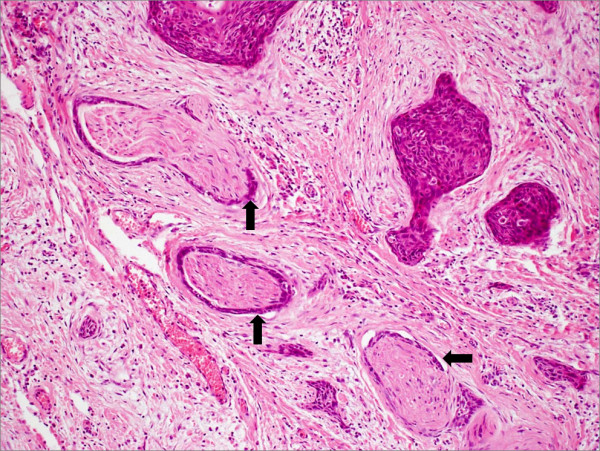
**HE stained section ×100 magnification showing nerve invasion**.

**Figure 17:**
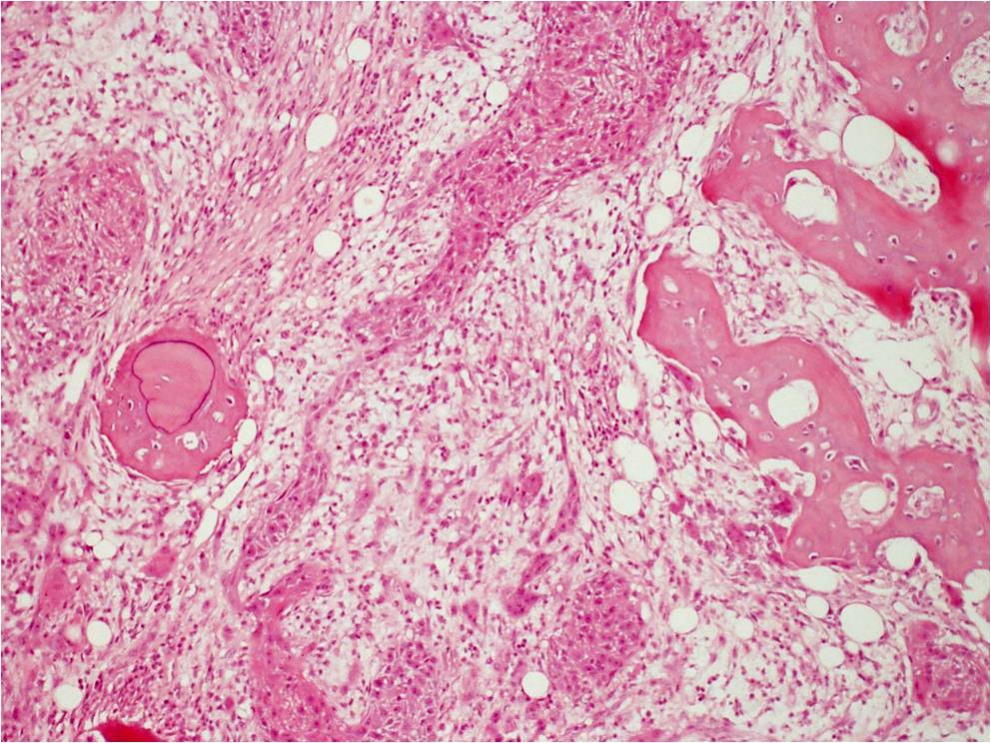
**HE stained section ×100 magnification showing bone invasion**.

Pathological analysis revealed that half of the patients had moderately differentiated OSCC, a quarter had well differentiated carcinoma and only 12 patients had poorly differentiated carcinoma (Figures 8, 9 and 10). pTNM differed somewhat from the cTNM and showed that only 70.4% of the patients had low-risk OSCC. Non-cohesive invasion (Figures 10 and 11) was reported in 33 patients, dysplasia at margin in 53 patients, and presence of severe dysplasia in 72 patients (Figure 12) with a mean depth of tumour invasion of 5.7 (SD3.8)mm (Figures 13 and 14). Vascular invasion was evident in 28 patients (Figure 15), while nerve invasion was identified only in 12 patients (Figure 16). Bone and/or cartilage invasion (Figure 17) was only present in 5 patients.

**Figure 18:**
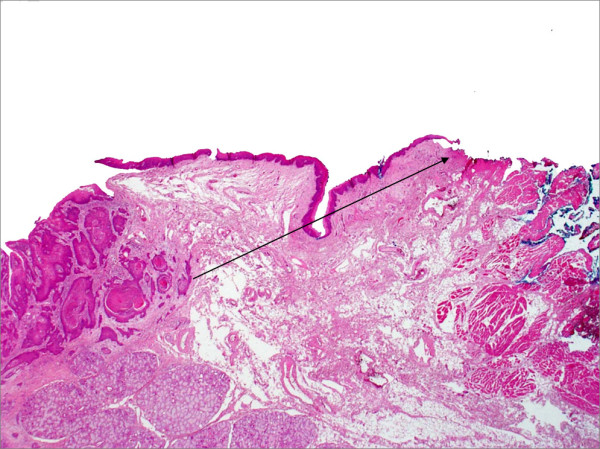
**HE stained section ×50 magnification showing Clear excision margin**.

Tumour clearance (Figure 18) was ultimately achieved in 107 (93%) patients; unfortunately, tumour recurred in 43 patients and was treated by further resection and/or radiotherapy. Other management modalities for recurrent disease included chemotherapy and photodynamic therapy. Follow-up resulted in a 3-year survival of 74.8% and a 5-year survival of 72.2% (Table 1).

Recurrence was identified in 23 males and in 20 females, with Caucasians being the most prominent group to report this (67.4%). The mean age of 1^st ^diagnosis of the recurrence group was 59.53 years. Most common oral sites included the lateral border of tongue (15) and floor of mouth (12). Recurrence was associated with clinical N-stage disease in 51.2% (p < 0.001) of the patients and pathological N-stage disease in 62.8% (p < 0.001) of the patients. Interestingly, 44.2% (p < 0.001) of the recurrences were in moderately differentiated OSCC. The histological sections in this group (n = 43) was evaluated and found that 17 had non-cohesive invasion pattern (p = 0.039), 30 had dysplasia at margin (p < 0.001), 21 had vascular invasion (p < 0.001), 9 had nerve invasion (p = 0.006) and 3 had bony invasion. Severe dysplasia was present in 37 patients (p < 0.001). Tumour clearance was previously achieved in only 8 patients (p < 0.001). The mean depth of tumour invasion for the recurrence group was 7.6 (SD3.8) mm (Table 2).

**Table 2:** Demographic details of 43 patients with recurrent oral squamous cell carcinoma

	**Recurr**.	Fisher's exact p-values		**Recurr**.	Fisher's exact p-values
**Gender**			**Differentiation**		
Male	23 (53.5%)		Well	8 (18.6%)	
Female	20 (46.5%)	0.377	Moderate	19 (44.2%)	
			Moderate-poorly	7 (16.3%)	
**Race**			Poorly	9 (20.9%)	<0.001
Caucasian	29 (67.4%)				
Indian	5 (11.6%)		**pTNM**		
Middle-Eastern	0 (0.0%)		T1N0M0	10 (23.3%)	
Oriental	0 (0.0%)		T2N0M0	6 (14.0%)	
Other Asians	2 (4.7%)		T1N1M0	4 (9.3%)	
African	5 (11.6%)		T2N1M0	4 (9.3%)	
Caribbean	2 (4.7%)	0.491	T1N2aM0	5 (11.6%)	
			T2N2aM0	7 (16.3%)	
**Primary site**			T1N2bM0	1 (2.3%)	
Floor of mouth	12 (27.9%)		T2N2bM0	3 (7.0%)	
Tongue (lateral)	15 (34.9%)		T1N2cM0	2 (4.7%)	
Tongue (dorsal)	2 (4.7%)		T2N2cM0	1 (2.3%)	<0.001
Tongue (ventral)	0 (0.0%)				
Buccal mucosa	2 (4.7%)		**IF**, cohesive	26 (60.5%)	
Hard palate	2 (4.7%)		**IF**, non-cohesive	17 (39.5%)	0.039
Upper alveolus	2 (4.7%)		**Dys. At Margin**	30 (69.8%)	<0.001
Lower alveolus	3 (7.0%)		**Lymphvascular Invasion**	21 (48.8%)	<0.001
Retromolar area	3 (7.0%)		**Nerve Invasion**	9 (20.9%)	0.006
Tuberosity	1 (2.3%)		**B/C Invasion**	3 (7.0%)	0.270
Upper lip	0 (0.0%)		**SD present**	37 (86.0%)	<0.001
Lower lip	1 (2.3%)		**Tumour clearance**	8 (18.6%)	<0.001
Neck Lump	0 (0.0%)	0.345			
			**Age at 1st OSCC**		
**cTNM**			Mean	59.53	0.703
T1N0M0	14 (32.6%)				
T2N0M0	7 (16.3%)		**Depth of Invasion (mm)**		
T1N1M0	1 (2.3%)		Mean	7.6	<0.001
T2N1M0	3 (7.0%)				
T1N2aM0	4 (9.3%)				
T2N2aM0	7 (16.3%)				
T1N2bM0	1 (2.3%)				
T2N2bM0	3 (7.0%)				
T1N2cM0	3 (7.0%)	<0.001			

**Figure 19:**
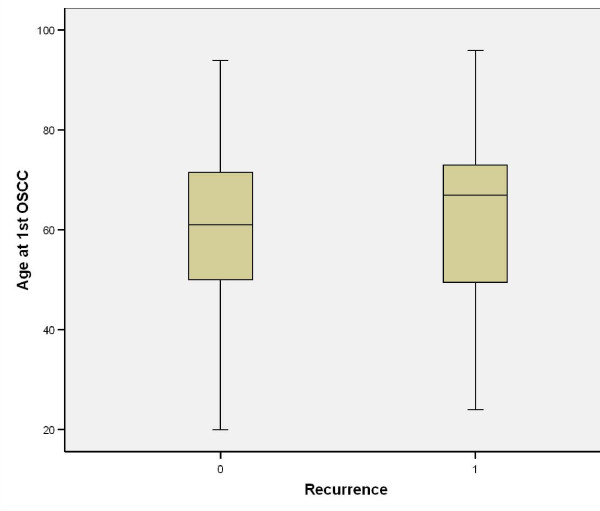
**Age at 1^st ^SCC vs. recurrence**.

**Figure 20:**
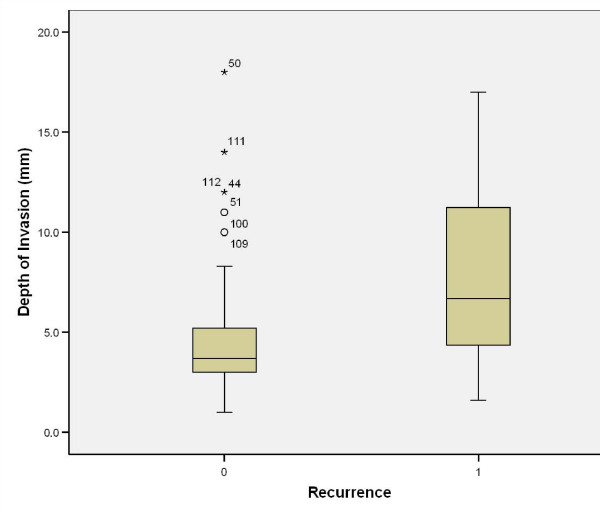
**Depth of tumour invasion vs. recurrence**.

Causes of death were either tumour related (i.e. locoregional or distant metastasis) or non-tumour related (e.g. pneumonia or any other cause that led ultimately to cardiorespiratory failure). An interesting finding was that 5/11 patients who died of distant metastasis had their primary disease in the tongue (p = 0.819). Nodal disease comparison showed that 8/10 patients who died of locoregional metastasis and 8/11 patients who died from distant metastasis had clinical nodal involvement (p < 0.001); (Table 3). On comparing this with pathological nodal disease it was noted that 10/10 patients and 10/11 patients who died from locoregional and distant metastasis, respectively, had nodal disease (p < 0.001). Tumour grading showed that half of the patients (5/10) who died from locoregional disease had poorly differentiated carcinoma (p = 0.001); interestingly 6/11 patients who died from metastatic disease had moderately differentiated OSCC (p = 0.001). Patients with recurrence were marginally older than non-recurrence patients (Figure 19). All patients who died from locoregional and distant metastasis were shown to have recurrence after the primary tumour resection (p < 0.001); (Table 4). The depth of invasion of tumour in recurrence patients was higher than non-recurrence (Figure 20).

**Table 3:** Gender, race, primary site and cTNM vs. cause of death

	Cause of death						Fisher's exact p-values
	**Alive (%)**	**Cardio-respiratory failure (%)**	**Pneumonia (%)**	**Regional met (%)**	**Distant met (%)**	**Total**	

**Gender**							
Male	50 (60.2)	4 (80.0)	0 (0.0)	5 (50.0)	6 (54.5)	65	
Female	33 (39.8)	1 (20.0)	6 (100.0)	5 (50.0)	5 (45.5)	50	0.039
							
**Race**							
Caucasian	56 (67.5)	4 (80.0)	5 (83.3)	6 (60.0)	7 (63.6)	78	
Indian	8 (9.6)	0 (0.0)	0 (0.0)	2 (20.0)	0 (0.0)	10	
Middle-Eastern	2 (2.4)	0 (0.0)	0 (0.0)	0 (0.0)	0 (0.0)	2	
Oriental	1 (1.2)	0 (0.0)	0 (0.0)	0 (0.0)	0 (0.0)	1	
Other Asians	4 (4.8)	0 (0.0)	0 (0.0)	0 (0.0)	2 (18.2)	6	
African	8 (9.6)	1 (20.0)	1 (16.7)	1 (10.0)	2 (18.2)	13	
Caribbean	4 (4.8)	0 (0.0)	0 (0.0)	1 (10.0)	0 (0.0)	5	0.914
							
**Primary site**							
Floor of mouth	16 (19.3)	3 (60.0)	3 (50.0)	0 (0.0)	2 (18.2)	24	
Tongue (lateral)	24 (28.9)	2 (40.0)	0 (0.0)	7 (70.0)	3 (27.3)	36	
Tongue (dorsal)	10 (12.0)	0 (0.0)	1 (16.7)	0 (0.0)	2 (18.2)	13	
Tongue (ventral)	5 (6.0)	0 (0.0)	0 (0.0)	0 (0.0)	0 (0.0)	5	
Buccal mucosa	7 (8.4)	0 (0.0)	2 (33.3)	1 (10.0)	1 (9.1)	11	
Hard palate	3 (3.6)	0 (0.0)	0 (0.0)	0 (0.0)	0 (0.0)	3	
Upper alveolus	4 (4.8)	0 (0.0)	0 (0.0)	1 (10.0)	1 (9.1)	6	
Lower alveolus	4 (4.8)	0 (0.0)	0 (0.0)	1 (10.0)	1 (9.1)	6	
Retromolar area	2 (2.4)	0 (0.0)	0 (0.0)	0 (0.0)	1 (9.1)	3	
Tuberosity	1 (1.2)	0 (0.0)	0 (0.0)	0 (0.0)	0 (0.0)	1	
Upper lip	1 (1.2)	0 (0.0)	0 (0.0)	0 (0.0)	0 (0.0)	1	
Lower lip	5 (6.0)	0 (0.0)	0 (0.0)	0 (0.0)	0 (0.0)	5	
Neck Lump	1 (1.2)	0 (0.0)	0 (0.0)	0 (0.0)	0 (0.0)	1	0.819
							
**cTNM**							
T1N0M0	54 (65.1)	2 (40.0)	2 (33.3)	1 (10.0)	3 (27.3)	62	
T2N0M0	20 (24.1)	1 (20.0)	2 (33.3)	1 (10.0)	0 (0.0)	24	
T1N1M0	3 (3.6)	0 (0.0)	0 (0.0)	0 (0.0)	0 (0.0)	3	
T2N1M0	2 (2.4)	1 (20.0)	1 (16.7)	1 (10.0)	0 (0.0)	5	
T1N2aM0	1 (1.2)	0 (0.0)	0 (0.0)	3 (30.0)	1 (9.1)	5	
T2N2aM0	0 (0.0)	1 (20.0)	1 (16.7)	2 (20.0)	5 (45.5)	9	
T1N2bM0	0 (0.0)	0 (0.0)	0 (0.0)	1 (10.0)	0 (0.0)	1	
T2N2bM0	1 (1.2)	0 (0.0)	0 (0.0)	1 (10.0)	1 (9.1)	3	
T1N2cM0	2 (2.4)	0 (0.0)	0 (0.0)	0 (0.0)	1 (9.1)	3	<0.001
							
**Total**	83	5	6	10	11		

**Table 4:** Differentiation, pTNM, invasive front, status of surgical margin and recurrence vs. cause of death

	Cause of death						Fisher's
	**Alive (%)**	**Cardio-respiratory failure (%)**	**Pneumonia (%)**	**Regional met (%)**	**Distant met (%)**	**Total**	

**Differentiation**							
Well	24 (28.9)	3 (60.0)	3 (50.0)	1 (10.0)	1 (9.1)	32	
Moderate	47 (56.6)	2 (40.0)	3 (50.0)	2 (20.0)	6 (54.5)	60	
Moderate-poorly	8 (9.6)	0 (0.0)	0 (0.0)	2 (20.0)	1 (9.1)	11	
Poorly	4 (4.8)	0 (0.0)	0 (0.0)	5 (50.0)	3 (27.3)	12	0.001
							
**pTNM**							
T1N0M0	53 (63.9)	2 (40.0)	2 (33.3)	0 (0.0)	1 (9.1)	58	
T2N0M0	20 (24.1)	1 (20.0)	2 (33.3)	0 (0.0)	0 (0.0)	23	
T1N1M0	4 (4.8)	0 (0.0)	0 (0.0)	1 (10.0)	1 (9.1)	6	
T2N1M0	2 (2.4)	1 (20.0)	1 (16.7)	2 (20.0)	0 (0.0)	6	
T1N2aM0	1 (1.2)	0 (0.0)	0 (0.0)	3 (30.0)	2 (18.2)	6	
T2N2aM0	0 (0.0)	1 (20.0)	1 (16.7)	2 (20.0)	5 (45.5)	9	
T1N2bM0	0 (0.0)	0 (0.0)	0 (0.0)	1 (10.0)	0 (0.0)	1	
T2N2bM0	1 (1.2)	0 (0.0)	0 (0.0)	1 (10.0)	1 (9.1)	3	
T1N2cM0	1 (1.2)	0 (0.0)	0 (0.0)	0 (0.0)	1 (9.1)	2	
T2N2cM0	1 (1.2)	0 (0.0)	0 (0.0)	0 (0.0)	0 (0.0)	1	<0.001
							
**IF**, cohesive	64 (78.0)	5 (6.1)	5 (6.1)	5 (6.1)	3 (3.7)	82	
**IF**, non-cohesive	19 (57.6)	0 (0.0)	1 (3.0)	5 (15.2)	**8 (24.2)**	33	0.002
							
**Dys. At Margin**	33 (62.3)	1 (1.9)	2 (3.8)	**8 (15.1)**	**9 (17.0)**	53	0.005
**Lymphvascular Invasion**	13 (46.4)	1 (3.6)	2 (7.1)	**6 (21.4)**	**6 (21.4)**	28	0.002
**Nerve Invasion**	5 (41.7)	0 (0.0)	0 (0.0)	**3 (25.0)**	**4 (33.3)**	12	0.011
**B/C Invasion**	2 (40.0)	0 (0.0)	0 (0.0)	1 (20.0)	2 (40.0)	5	0.131
**SD present**	49 (68.1)	3 (4.2)	3 (4.2)	7 (9.7)	10 (13.9)	72	0.271
**Tumour clearance**	82 (76.6)	5 (4.7)	5 (4.7)	6 (5.6)	9 (8.4)	107	<0.001
							
**Recurrence**	19 (44.2)	2 (4.7)	1 (2.3)	**10 (23.3)**	**11 (25.6)**	43	<0.001
							
**Total**	83	5	6	10	11		

**Figure 21:**
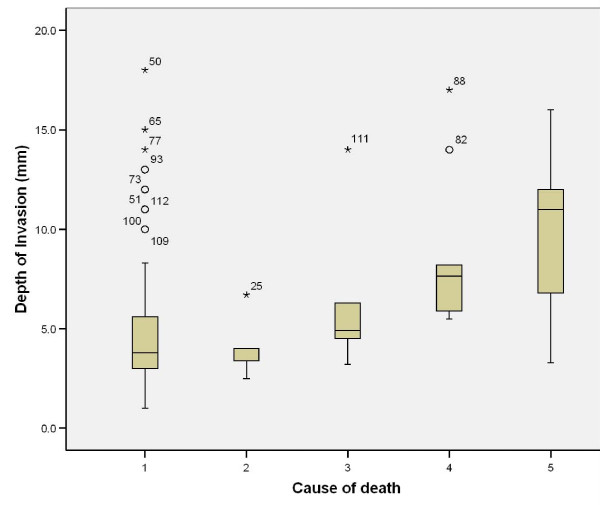
**Age at 1^st^ SCC vs. survival**. (1) Alive, (2) death from any non-tumour event that lead to cardiorespiratory failure, (3) death from bronchopulmonary pneumonia, (4) death from locoregional spread, and (5) death from distant metastasis.

Further analysis of pathological variables in relation to cause of death revealed that non-cohesive invasion is linked to death from distant metastasis, when compared to cohesive invasion (p = 0.002); dysplasia at margin indicates poor prognosis and death from locoregional and distant metastasis (p = 0.005), however presence of severe dysplasia was not significantly related to tumour-related death. Tumour capability to invade nerves and vessels carried poor prognosis with p = 0.011 and 0.002, respectively, but this was not the case with bone and cartilage invasion (p = 0.131). The presence of positive margins, even with subsequent radiotherapy, carried high risk of death from locoregional and distant metastasis (p < 0.001); (Table 5); similarly, this was the case in tumour depth of 8.6 (SD3.8)mm for locoregional spread and 9.5 (SD3.7)mm for distant spread (Table 5), (Figure 21).

**Table 5:** Age at 1^st ^OSCC and depth of invasion vs. cause of death

	Cause of death					Kruskal wallis p-values
	**Alive at 5-years**	**Cardio-respiratory failure**	**Pneumonia**	**Regional met**	**Distant met**	

**Age at 1st OSCC**						
Mean	58.73	85.20	83.83	67.70	55.82	<0.001
Std. Deviation	14.373	8.228	7.026	12.230	15.276	
Std. Error	1.578	3.680	2.868	3.867	4.606	
Lower Bound 95% CI	55.60	74.98	76.46	58.95	45.56	
Upper Bound 95% CI	61.87	95.42	91.21	76.45	66.08	
Minimum	20	73	73	49	34	
Maximum	91	96	94	85	72	
						
**Depth of Invasion (mm)**						
Mean	4.837	4.000	6.300	8.620	9.545	<0.001
Std. Deviation	3.4516	1.6016	3.9085	3.8250	3.7377	
Std. Error	0.3789	0.7162	1.5956	1.2096	1.1270	
Lower Bound 95% CI	4.084	2.011	2.198	5.884	7.034	
Upper Bound 95% CI	5.591	5.989	10.402	11.356	12.057	
Minimum	1.0	2.5	3.2	5.5	3.3	
Maximum	18.0	6.7	14.0	17.0	16.0	

**Figure 22:**
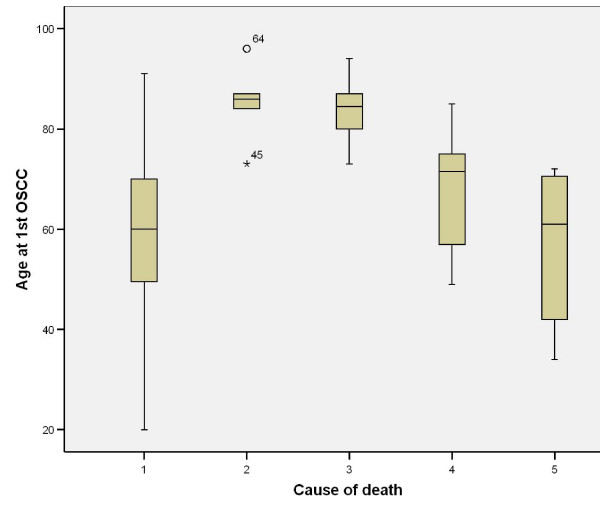
**Depth of tumour invasion vs. survival.** (1) Alive, (2) death from any non-tumour event that lead to cardiorespiratory failure, (3) death from bronchopulmonary pneumonia, (4) death from locoregional spread, and (5) death from distant metastasis.

Cause of death vs. patient's age revealed that older patients are more likely to die from bronchopulmonary pneumonia or any non-tumour sequel which results in cardiorespiratory failure (Table 5), (Figure 22).

**Figure 23:**
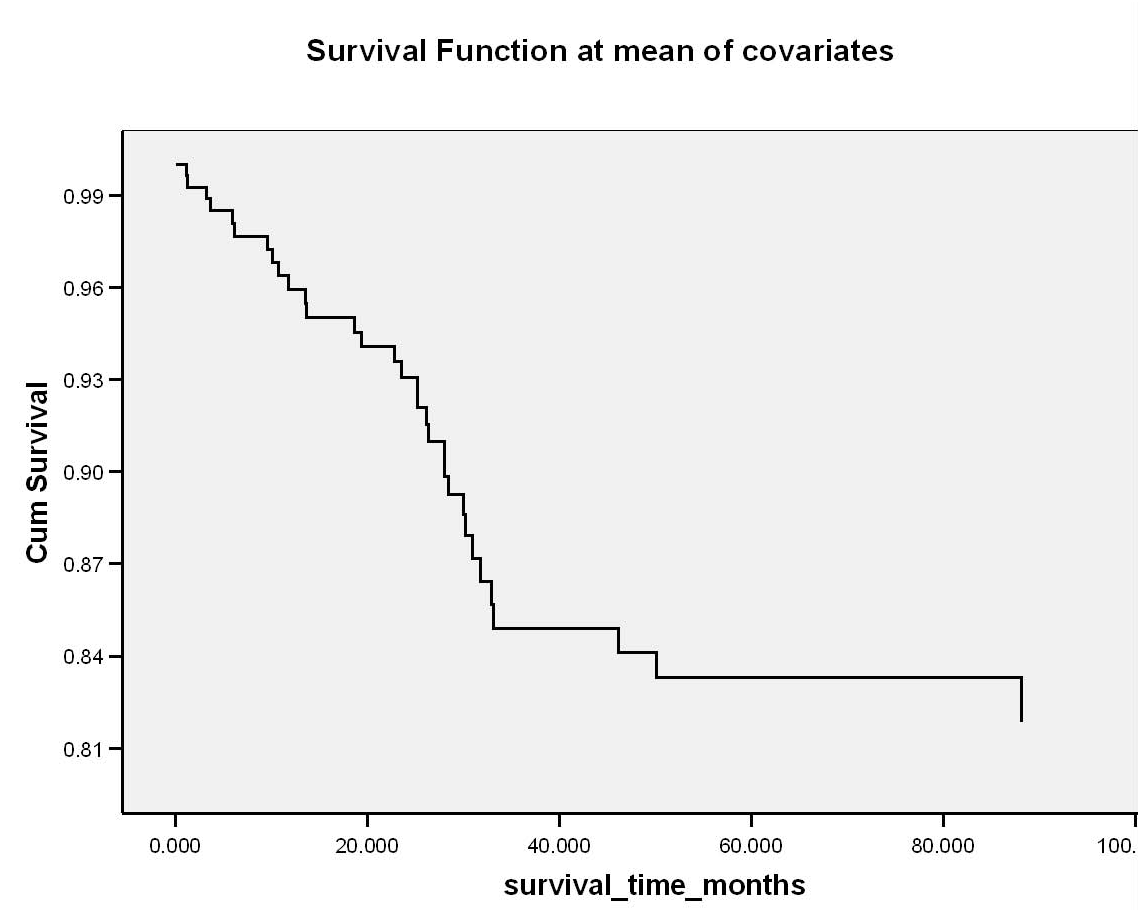
**Kaplan Meir survival calculations per demographic and tumour factor assessed**.

Logistic regression analysis on all the overall clinicopathological variables as well as the numerical covariates revealed that age at 1^st ^OSCC significantly affected survival at 3-years and at 5-years (p = 0.001); grading (pTNM) was found to be significant at 3-years (p = 0.008) and 5-years (p = 0.025); (Table 6). Kaplan-Meir (survival) curve is illustrated in figure 23. Cox regression analysis reported significance in age at 1^st ^SCC (p = 0.001; Exp B = 1.057) and grading (pTNM) (p = 0.001; Exp B = 2.914).

**Table 6:** Logistic regression analysis on all the overall clinicopathological variables

	3-years		5-years	
	odds ratios	p-values	odds ratios	p-values
Age at 1^st ^OSCC	1.085	**0.001**	1.096	**0.001**
Gender	5.504	**0.025**	3.252	0.111
Grading cTNM	0.527	0.345	0.843	0.806
Differentiation	1.111	0.794	0.994	0.990
Grading pTNM	8.012	**0.008**	5.707	**0.025**
Invasive front-invasion	0.891	0.894	1.374	0.713
Dysplasia at margin	0.361	0.259	0.244	0.130
Lymphvascular invasion	2.067	0.425	3.508	0.174
Nerve invasion	1.460	0.752	1.723	0.671
Tumour clearance	0.387	0.411	0.200	0.233
Recurrence	2.211	0.383	3.137	0.207

## Discussion

The aim of surgical ablation for oral squamous cell carcinoma is the removal of all viable tumour tissue. This intuitively is associated with better overall prognosis. Occasionally despite the small tumour dimensions (as in early disease), the actual biological characteristics of the cancer result in residual disease despite good clearance because of the existence of suppressed *tumour clonogens *which activate after removal of the main tumour mass. This provides some explanation as to why occasionally indolent seeming lesions undergo massive local recurrence after removal of the primary lesion. Several clinicopathological parameters are being discussed in relation to incidence, recurrence, disease progression and survival.

### I. Gender

Oral cancer is known to affect more males than females with an approximate ratio of 1.5:1, respectively. Nearly a quarter of the newly diagnosed cancers in males from Sri Lanka, India, Pakistan and Bangladesh are located in the head and neck region [5,6]. The male:female ratio in our study was 1.3:1. Recurrence of the disease was identified in 23/65 males and 20/50 females.

Male patients who died from non-tumour causes were more likely to suffer cardiorespirtory failure; while female patients died from bronchopulmonary pneumonia (p = 0.039). The gender factor was not significant when comparing death from locoregional or distant metastasis.

### II. Age at 1st SCC

United States (SEER) data reported that the large majority of OSCC patients are over 45 years of age, with a median age of 1^st ^SCC diagnosis at 62 years [7].

About 6% of oral cancers occur in young people under the age of 45 years [8]. Young age in patients with SCC of the tongue appeared to be an independent predictor of worse survival in another study [9], but a further study comparing the relative survival of young people (under 45 years of age) with oral cancer compared with the survival of older people (45 years and older) showed a higher 5 year relative survival among young people compared with the older group [10]. Younger patients usually report problems with appearance after cancer treatment [11].

In our study, the youngest patient was diagnosed at age of 20 and the oldest at 96; mean diagnostic age was 61.70. Mean recurrence age was 59.53. Age at 1^st ^SCC is a very significant predictor for survival at 3 and 5 years. Older patients tend to die from cardiorespiratory failure (mean 85.20 years) and bronchopulmonary pneumonia (mean 83.83). Patients who died from distant metastasis (mean 55.82 years) are younger than those who died from locoregional tumour spread (mean 67.70 years). Logistic regression analysis revealed that younger patients have worse prognosis.

### III. Race

South and Southeast Asia (i.e. Sri Lanka, India, Pakistan and Taiwan), Latin America and the Caribbean (i.e. Brazil, Uruguay and Puerto Rico), Pacific regions, Eastern Europe (i.e. Hungary, Slovakia and Slovenia) and some parts of the Western world (i.e. France) are characterised by high incidence rates for oral SCC [5,6].

Tongue SCC is significantly higher in Blacks compared to Whites within the same regions of the United States. The prevalence of oral cancer is also generally higher in ethnic minorities in other developed countries [12,13].

A recent, interesting, oral cancer survival study comparing British South Asian population of South-East England to the Non-South Asian population showed that South Asian males have significantly better survival than their Non-South Asian peers [14].

Two-thirds of our patients were Caucasians due to the geographic distribution of the population in the area. Only 19 patients were from Asian background. Recurrence of the disease was identified in 29 Caucasians (67.4%), 5 Indians (11.6%), 5 Africans, 2 Caribbeans (4.7%) and 2 of other Asian origin. When comparing patient's race and cause of death no significance was identified. The majority of death was among Caucasians as they represent 67.8% of the study population. An interesting finding was that 3 out of the 13 African patients died from tumour-related causes (one from locoregional metastasis and two from distant metastasis).

### IV. Primary site

The most commonly reported oral cancer sites include the floor of the mouth (FOM) and lateral borders of the tongue. The tongue, as a whole, is the most common (40-50%) site for oral SCC in European and American population. Asian population usually suffer from cancer of the buccal mucosa due to betel quid/tobacco chewing habits; Buccal mucosa SCC constitute 40% of OSCC in Sri Lankan population [13].

Five-year survival is significantly reduced for more posteriorly located tumours (i.e. oropharyngeal compared to oral) [15]. Reduction in survival is largely explained by tumour's site influence on nodal metastasis [16]. The surgeon's ability to achieve clear resection margins may be restricted by accessibility to the tumour's primary site and the need for adjuvant therapy postoperatively (i.e. radiotherapy).

In our study, the majority of our patients suffered from tongue cancer (n = 54) and FOM cancer (n = 24). Recurrence was associated with primary tumours of the tongue (34.9%) and floor of mouth (27.9%). High association was identified between tumour-related death and location of primary. 7/10 dead patients from locoregional metastasis suffered from SCC of lateral border of the tongue and 7/11 patients who died from distant disease suffered FOM and tongue SCC; this indicates that site of primary can predict prognosis; this can be linked to the lymphatic drainage of these locations (via the deep cervical chains).

### V. Tumour size and thickness (depth of invasion)

The tumour size usually affects choice and outcome of treatment [15]. It also affects the surgeon's ability to achieve complete resection, especially in deep invading tumours. Increased tumour size has been linked to cervical involvement [16-18], high recurrence rate [16,19,20] and poor prognosis [21,22]. However, a recent study suggested that tumour size did not predict nodal disease [23].

A precise clinically optimal tumour thickness cut-off point has not been established [24]. The cut-off thickness varies from centre to centre. The association of tumour thickness with lymph node metastasis is believed to reflect the aggressiveness of tumour growth [25].

Sixteen relevant studies were examined for the cut-off tumour thickness points (3,4,5 and 6 mm); there was a statistically significant difference between the 4 mm and 5 mm tumour thickness cut-off points and cervical lymph node involvement in OSCC [24].

It has been suggested that a high relationship exists between tumour thickness and ipsilateral cervical metastasis [26-30]. The relationship between thickness of the primary tumour and occurrence of contralateral cervical metastasis were reported to increase by 5% in T1/T2 SCC of the oral tongue [31]. It is now widely accepted that thickness is more accurate predictor of sub-clinical nodal metastasis, local recurrence and survival than tumour size [16].

In our study, the mean tumour depth was 5.7 mm ± 3.8, with a maximum registered depth of 18 mm. Mean depth of invasion in disease recurrence was found to be 7.6 mm ± 3.8. Death within 3 years of diagnosis was related to tumour depth (p = 0.043), however on further follow-up it was found to be insignificant. Alive patients at 5 years registered a tumour depth of invasion of 4.8 mm ± 3.5, compared to 8.6 mm ± 3.8 for patients who died from locoregional spread and 9.6 mm ± 3.7 for those who died from distant disease. Tumour depth of invasion is a good prognostic indicator.

### VI. Nodal involvement and TNM system

This continues to be an interesting topic for oncology surgeons; incidence of ipsilateral, contralateral or bilateral nodal involvement has been studied. Worse prognosis is expected in patients with nodal disease [32]; this worsens with the presence of extracapsular spread [33]. The incidence of occult lymph node metastasis in early stage tumours (T1/T2) has been reported to be between 27%-40% [34-36].

Obviously, the status of ipsilateral neck is important in assessing the risk to the contralateral neck; in one study 22% false-negatives were quoted on contralateral assessment [37]; another study reported 10% [31]. Extracapsular spread was identified as an important predictor of regional recurrence, distant metastasis, and thus, overall survival [38].

Factors that seem to influence tumour spread to the lymphatics include tumour primary site, thickness, double DNA aneuploidy and poor differentiation [26,31]. Other identified factors include peri-neural invasion, infiltrating-type invasive front and T2 tongue tumours [29], as well as low E-cadherin for prediction of late cervical metastasis [30].

Distant metastasis was reported to occur in 5-25% of OSCC patients [39], most commonly in uncontrolled locoregional and N-stage diseases, especially N2/N3. Extracapsular spread is a very strong predictor for systemic spread [16,38,40].

The TNM classification of the International Union Against cancer (UICC) relates well to the overall survival [11,15]. The earlier the tumour stage, the better the prognosis and the less complicated is the treatment [41]. There is a growing concern that TNM staging is insufficient to accurately map or classify OSCC, whose biological impact may be related to volume and pathological aggressiveness of disease.

Tumour diameter or surface greatest dimension is used to indicate tumour size in the TNM system [42]; however, this is not the most accurate when compared to tumour thickness or depth of invasion, which can be related directly to prognosis [[16,43-45]).

In our study, nearly 75% were diagnosed with T1/T2 N0 tumours, 8 patients had N1 disease and 21 had N2 disease. Pathological confirmation showed that 12 patients had N1 disease and 22 patients had N2 disease. Recurrence was mainly associated with N-stage disease; clinically 41.9% of the recurrences had N2-stage disease, while pathologically this was evident in 44.2% of the patients. All the patients (10/10) who died from locoregional disease had nodal involvement, with the majority being N2 (7/10 patients); while 10/11 patients with nodal involvement died from distant metastasis, with the majority being N2a (7/10 patients). A haematogeneous tumour spread has been suggested with regard to the one patient who had no nodal disease but died of distant metastasis. Logistic regression analysis revealed that the worse the pTNM, the worse the prognosis. TNM system is a good indicator of tumour prognosis.

### VII. Differentiation

It is widely accepted that prognosis is better in early cancers, particularly those that are well-differentiated [11,15]. The WHO grading system [46] recommends 3 categories: well differentiated, moderately differentiated and poorly differentiated. This usually depends on the subjective assessment of the degree of keratinisation, cellular and nuclear pleomorphism, and mitotic activity [16]. The influence of histologic grading as a prognostic factor in OSCC was assessed in 215 patients and was found to be a significant predictor of locoregional failure and tumour recurrence [47]. Multivariate analysis study showed that tumour grade was significantly related to nodal disease at the time of diagnosis [23]; however most authorities consider this grading system as a poor indicator of outcome and response to treatment [16,44,46,48].

In our study, half of the patients had moderately-differentiated SCC and about 10% had poorly-differentiated tumour. Recurrence was mainly associated with moderately differentiated tumours. 9/10 patients who died of locoregional spread had moderately, moderate-poorly and poorly differentiated SCC; 5/9 had poorly-differentiated tumour (p = 0.001). 10/11 patients who died of distant disease suffered from moderately, moderately-poorly and poorly differentiated SCC, with 6/10 of these having moderately-differentiated tumour (p = 0.001).

### VIII. Invasive front (IF)-pattern of invasion

An extensive review of the impact of invasive front is beyond the scope of this manuscript. The invasive front (tumour cells at the most invasive part of the malignant tumour) differs significantly from the central or superficial part of the tumour [49]. Understanding the biological behaviour of these cells has lead to the link between these cells and the risk of cervical metastasis in OSCC patients [50]. Image and flow cytometric analysis of the invasive front cells showed abnormal DNA content (4cER), thereby confirming that this can give additional useful information when selecting treatment strategies [3].

There is technical and logistic difficulty in assessing the invasive front which if performed rigorously allowed authorities to differentiate between epithelial dysplasia, carcinoma in-situ and invasive cancer [11,15,16].

The pattern of invasion can be assessed by using Anneroth et al. and Bryne et al. criteria. Grade 1 tumours had well-delineated "pushing or cohesive" borders. In Grade 2, the advancing edge of tumour infiltrated in solid cords, bands or strands. Grade 3 tumours had margins that contained small groups or cords of infiltrating cells. In Grade 4, there is marked dissociation in small groups or even single cells (non-cohesive) [51].

Endophytic growth pattern is associated with increased local recurrence. High grades of infiltration (grade 3 or 4) are usually associated with nodal involvement and subsequent disease metastasis; while this was not associated with local recurrence. Pattern of invasion didn't affect cumulative survival [51]. Another study on 68 OSCC patients confirmed that the pattern of invasion was not significantly related to local recurrences [52].

In our study, pathology reports showed that 33 patients had non-cohesive pattern of invasion. In recurrence states, non-cohesive invasion was identified in 17 patients, while cohesive fronts were evident in 26 patients. 5/33 patients with non-cohesive invasion died from locoregional spread; while 8/33 died from distant metastasis (p = 0.002). This suggests that non-cohesive invasion is a significant prognostic factor associated with distant disease [53].

### IX. Presence of severe dysplasia (SD) and dysplasia at margin

There are variations in the pathological interpretation and classification of dysplasia. It is widely accepted that dysplasia precedes OSCC [54] and that 11% of OSCC patients had cancer elsewhere [55]. Field cancerisation concept and the presence of dysplastic epithelium in cancerous tissue have been reported in a number of studies [56,57].

A study on small group of patients has revealed that the presence of mild or moderate epithelial dysplasia at the margins of surgically removed OSCC carries a significant risk for the development of local recurrence [58]; it is worth noting that patients with severe dysplasia were excluded from the study as it was believed that the pathology overlaps with carcinoma in situ.

In this study, severe dysplasia was present in the pathology specimens of 72 patients and dysplasia at margin was identified in 53 patients. Recurrence was seen in 37/43 with severe epithelial dysplasia and in 30/43 patients with dysplasia at margins. Severe dysplasia was present in the specimens of 7/10 and 10/11 patients who died from locoregional spread and distant metastasis, respectively (p = 0.271). Dysplasia at margin was identified in the surgical resection of 8/10 and 9/11 patients who died from locoregional spread and distant metastasis, respectively (p = 0.005). Dysplasia at margin is an excellent predictor of tumour spread.

### X. Lymphvascular and nerve invasion

Lymphvascular and peri-/endoneural invasion show a significant association with tumour size, histological grading, invasive front, nodal involvement, status of the surgical margins, overall prognosis and survival [15].

Lymphvascular invasion implies a considerable number of tumour cells are entering the vascular compartment which increases the likelihood of regional and distant metastasis [16,59].

A recent study reported that a weak or limited lymphocyte response at the tumour/host interface is strongly associated with local recurrence and death [53]. An inverse relationship was also reported by other studies, between lymphocytic infiltrate and nodal disease and overall prognosis [60,61].

It has been proposed that tumour emboli are more difficult to form in the small-calibre lymphatics of superficial areas than in the wider lymphatics of deep tissue, hence tumour thickness may play a vital role in lymphvascular invasion [24,62].

In this study, vascular invasion was reported in 28 patients. Recurrence was detected in 21/43 patients with lymphovascular invasion. Out of 28 patients with lymphovascular invasion 6 died of locoregional metastasis and 6 died of distant metastasis (p = 0.002). This indicates that this is one of the determinant factors in prognosis.

Prognostic value of perineural invasion has been highlighted in several studies and linked to regional recurrence and distant metastasis [63,64]. Others detected no such association [52].

In a recent multivariate analysis of perineural invasion of small and large nerves, invasion of large nerves was associated with local recurrence [53].

In this study, perineural invasion was reported in 12 patients. Recurrence was seen in 9/43 patients who reported this invasion. 3/12 and 4/12 patients with lymphovascular invasion died from locoregional and distant metastasis, respectively (p = 0.011). This indicates that this is another determinant factor in prognosis.

### XI. Bone/cartilage (B/C) invasion

Bone and cartilage invasion affect prognosis [11,15]. This usually influences the type and extent of treatment [16]. Extensive work in this area has been carried out by Julia Woolgar who suggested that T4N0 have a better prognosis than the other stage IV categories.

In our study, only 5 patients were reported to have invasion of the mandibular cortical plate. Three of those patients reported disease recurrence. 1/5 and 2/5 patients with bone invasion died from locoregional and distant metastasis, respectively (p = 0.131).

### XII. Tumour clearance

The UK guidelines consider both mucosal and deep margins of 5 mm and more as clear, 1-5 mm as close and less than 1 mm as involved [16,65]. This usually ignores the formalin-shrinkage effect which can be at least 30% [51]. So in order to achieve a 5 mm pathological clearance, 8-10 mm in situ surgical margin need to be taken [66].

Positive or close margins are associated with increase in local recurrence [51] and have a negative effect on survival [67,68]. Furthermore, several studies have shown that local recurrence and overall survival benefit from achieving negative resection margins [51,69-71].

Interestingly, a study revealed that the presence of tumour cells within a distance of less than 5 mm, but not into the deep surgical margin, does not necessarily require additional treatment [52].

Despite the use of intraoperative frozen section analysis, 7% of our patients had close or positive margins on final histologic sections. This compares favourably with the world's literature [47,72,73]. When assessing disease recurrence, 8/43 patients had clear margins at the primary resection; this suggests high-risk surgical margins (i.e. non-cohesive, lymphovascular involvement) and biologic or genetic characteristics as the likely cause. Number of deaths in patients with locoregional and distant metastasis exceeds the number of deaths in patients with positive margins; this indicates that recurrence and tumour progression are possible even when achieving clear (tumour-free) margins [53].

### XIII. Management

Currently the gold standard management is surgery. Radiotherapy has been proposed as neo-adjuvant and adjuvant with chemotherapy. Photodynamic therapy is moving towards becoming the "fourth modality"; favourable results have been achieved in managing advanced tumours of the head and neck, using PDT.

Patients with nodal recurrence have a significantly worse disease free survival compared to patients without [11,15]. Pathological extent of the metastatic disease at the time of initial surgery tends to influence the rate of recurrence [16,74,75]. Others include surgical intervention and adjuvant therapy [38,40]. Survival is better in patients with local recurrence versus regional recurrence [76]. The reported mean survival following distant spread is less than 6 months and 90% of the cases are dead by 2 years [39].

#### XIIIa. Surgery

Surgery continues to be the well established mode of initial definitive treatment for the majority of OSCC patients [77]. Resection of the primary tumour is employed with dissection and removal of the cervical lymphatic chain, when indicated. Reconstruction of the defect can be by locoregional repair or by distant free tissue transfer. The employment of free tissue transfer combined with radiotherapy has improved survival from 40% to 70% [78].

Elective neck dissection is employed when the risk of cervical involvement is over 15-20% [24,79,80]. Elective neck dissection may be both diagnostic and therapeutic. It helps in defining the status of the neck, removal of undetectable metastasis and determines the need for adjuvant therapy [24]. Therapeutic neck dissection is of high benefit in patients with regional metastasis and has also been of benefit in patients with N0 neck [81,82]; however controversies arise in patients with T1N0 disease. Aggressive adjuvant therapy has been recommended for patients with extracapsular spread [38].

The most commonly used flap includes radial forearm, mandibular fibula free flap reconstruction, deep circumflex iliac artery and perforators. Oral oncologic reconstruction showed that the submental artery island flap is simple and reliable [83]; the jejunum flap after circumferential pharyngolaryngectomy has a high success rate [84].

In this study, management of the primary tumours was with surgery (n = 90), surgery followed by radiotherapy (n = 22) and surgery with chemoradiotherapy (n = 3). Few of the recurrences were treated with surgery (n = 2) and surgery with radiotherapy (n = 13). Surgery involved primary tumour resection. When there was a nodal disease, neck dissection and free tissue transfer was employed. Management of recurrence was mainly by radiotherapy (21/43), which was sometimes preceded by surgery (13/43). Surgery alone was given to 2 patients.

#### XIIIb. Chemoradiotherapy

Radiotherapy plays a key role in the management of early-stage and locally advanced SCC, either alone or more frequently combined with surgery and/or chemotherapy [1,85]. Postoperative radiation effect is the reason why positive tumour margins are controlled locally [51].

The role of chemotherapy in the management of OSCC continues to evolve. Locoregional advanced SCC can respond to chemotherapy, as an induction or palliative treatment, with irradiation. The current most favoured regimens for induction chemotherapy include cisplatin/infusional 5-fluorouracil/docetaxel [86].

Recent trials have showed that the use of concurrent single agent chemoradiotherapy (cisplatin) lead to a clear survival benefit of 11% [87,88].

In this study, management of the primary tumours was with surgery followed by radiotherapy (n = 22) and surgery with chemoradiotherapy (n = 3). Few of the recurrences were treated with surgery (n = 2) and surgery with radiotherapy (n = 13). Most recurrences were treated with radiotherapy (n = 21), surgery and radiotherapy (n = 13) and chemoradiotherapy (n = 5).

#### XIIIc. Photodynamic therapy (the "fourth modality")

Photodynamic therapy (PDT) is a minimally invasive method of treating a variety of tumours. The treatment can be delivered under local or general anaesthesia, and the delivery method includes surface illumination or interstitial application (iPDT). This therapy can be repeated as required as there is no cumulative toxicity; it can also be applied before or after any of the conventional treatment modalities. In this study, two patients with recurrent disease underwent photodynamic therapy.

The management of patients with premalignant lesions of the oral mucosa in "field cancerisation", with multicentric foci of invasion, presents a considerable problem for the surgeon. One study reported the use of PDT to treat 11 patients with "field cancerisation" occurring in the oral cavity, with excellent outcome [89]. Nineteen patients with histologically confirmed oral cancer (8 with field change disease) and one with severe dysplasia, were sensitized and treated with mTHPC-PDT. The results were assessed clinically and histologically. Most patients healed very well, but tongue tethering was seen in 1 patient and another had necrosis in normal areas due to light scattering within the mouth [90].

A phase I-II study was conducted to assess the safety and efficacy of iPDT for patients with persistent or recurrent head and neck cancer unsuitable for further treatment with surgery, radiotherapy or chemotherapy, recruited for 'last hope' salvage treatment. The results showed that 9 patients achieved a complete response and five are alive and free of disease 10-60 months later. The median survival was 16 months for the 33 responders, but only 2 months for the 12 non-responders [91].

### XIV. Morbidity and mortality

True recurrence develops much earlier than metachronous disease and carries the worst prognosis [16,92]. One study reported that 20/200 patients reported true recurrence and 18/20 died from the disease. While only 4/15 patients died of the disease [17]. Table 2 shows that in our study of early tumours the biology of the lesion and the histopathology of its excision (i.e. the margin) were significant indicators of recurrence.

To clarify the use of terms in the tables 3 and 4: we use 3-year and 5-year survivals to try and allow comparison between patient groups. However there are problems with the exact timing used in published studies creating a 'lead time bias' effect often which confounds analysis if not explicitly stated, suggesting an erroneously beneficial effect. Biologically these timing do not reflect tumour doubling but only current medical convention and separate timing for each specific pathology may be more valuable i.e. for instance 30 months survival is important in oral squamous cell carcinoma since most recurrences tend to occur before this time. These empirically derived year's survival figures are however a useful rule of thumb for quick review where 3 year survival reflects recurrence and 5 year survival reflects the modality used and overall pathology. In head & neck squamous cell cancer with its usual stepwise progression, locoregional failure is an outcome to be avoided. It is conventional wisdom that distant metastasis may have occurred at a very early stage in tumour growth and may not have been easily identified and managed by locoregional treatment i.e. surgery or radiotherapy and so is conveniently de-emphasized when comparing treatments as unavoidable, recent advances in molecular biology has highlighted this perception as erroneous. We also need to consider our patient populations (Table 5) with their significant co-morbidities (some of which have a common origin in the cancer aetiology i.e. smoking and alcohol) which themselves have a major impact on tumour treatment and patient survival (i.e. hypoxia reducing radiotherapy effect or atherosclerosis reducing anastomosis viability). This lends itself to the statistical iteration of eliminating the effect of non-tumour associated deaths for modality comparison; however logically and holistically this again is a conventional often used statistical distortion since the tumour/host is represented by one entity, the patient. It may be used to help economic arguments when considering the cost/benefit of treatments when results of unprocessed data fail to produce clarity.

### XV. Multidisciplinary approach and ethical considerations

Modern management of head and neck cancer is almost universally coordinated through a multidisciplinary team. The team consists of surgeons, medical oncologists, radiation oncologists, pathologists, AHPs (Allied Health Professionals) and radiologists. The discussions within this group are variable in their nature and transparency. It is not unusual for one particular group to dominate the views of the MDT. Often, very strong views are held by different specialties with some surgeons willing to operate on almost anyone, while radiation oncologists might believe that a primary chemo-radiotherapeutic approach is better for the patient. The final discussion with the patient is similarly biased towards the speciality of the person giving the advice and almost any decision can be justified by saying that it is the patient's choice. However, that is not to say that patients should not have a choice. Their views are paramount especially if they are provided with good quality honest information about survival rates with different treatment approaches and issues about quality of life.

Most research in head and neck cancer is targeted at evaluation of new chemotherapeutic agents. To date, this approach with at least recurrent disease has been remarkably unrewarding. However, with pressure to recruit to trials, patients will often be directed down the path of an industrially funded chemotherapy trial as opposed to being offered more conventional treatment. With management of head and neck, it has been clearly shown that unless one gets a complete response, the patient's prognosis is poor. Most drug trials talk in terms of overall response with complete response rates being usually in single figure percentages. So it is justifiable to question the ethics of many drug trials in advanced head and neck cancer.

**Management of T1 disease: **Most people would agree that T1 lesions can be treated by simple excision. This can be achieved either with a scalpel or with a laser. The advantage of using a laser is a relatively bloodless operation and less scar tissue formation due to reduced myofibroblastic contraction. Photodynamic therapy has also been used effectively in the management of T1 disease. Management of the neck is somewhat more controversial. Until recently, tumour thickness has been the most accurate predictor of metastatic lymphadenopathy with a cut-off at about 4-6 mm being indicative of an increased risk of neck metastasis. This has been the basis of the SEND trial which seeks to identify patients who benefit from elective neck dissection in T1/early T2 disease.

Much of this has been rendered unnecessary by the use of ultrasound investigation of the neck usually in conjunction with ultrasound guided fine needle aspiration cytology. This approach gives high sensitivity and specificity in evaluation of the neck. Even in the management of the neck when a primary neck dissection is not performed, interval ultrasound scanning would appear to pick up metastatic nodal disease before extranodal spread has occurred.

The use of very accurate surgically directed radiotherapy in the form of brachytherapy for very early disease may be justified in some cases i.e. where the surgical sequelae outweigh the disadvantage of using a modality which in essence can only be used once. We must consider carefully the bystander tissue irradiation which may have a significant adverse host effect when treating localised early cancer [93]. Very early lesions in the form of T1/early T2 disease are more appropriately surgically treated with an adequate margin of surgical excision with perhaps adjunctive therapies used at the margin of the lesion in cases of adverse histopathological features. Electroporation to the margin may also be considered in selected cases [94]. Both modalities are limited by availability and brachytherapy in particular by the additional patient burden in the form of a period of isolation for radiation protection purposes.

**Management of T2 disease:** This is even more controversial. A true thin T2 lesion can probably be safely resected but as soon as there is any increase in tumour thickness, neck dissection would be advocated. At this stage with thicker T2s and as well as T3 and T4, the size of the elective surgical defect would make reconstruction necessary. To some extent, this obviates any discussion about the need for neck dissections as this will be incorporated in the management.

In summary, squamous cell carcinoma of the oral cavity has a poor overall prognosis with a high tendency to recur at the primary site and extend to involve the cervical lymph nodes. In this article we have discussed several clinicopathological parameters that can be utilised to predict outcome, recurrence and overall survival.

## Competing interests

The authors declare that they have no competing interests.

## Authors' contributions

WJ: designed the study, carried out data collection, literature research, manuscript preparation and manuscript review. TU: designed the study, carried out data collection, literature research, manuscript preparation and manuscript review. AP: contributed to conception and design, carried out manuscript preparation and manuscript review. Also, carried out the statistical analysis. AR: carried out the literature research, data collection and manuscript preparation. ZH: carried out the literature research, data collection and manuscript preparation. MV: carried out the literature research, data collection and manuscript preparation. KK: carried out the literature research, data collection and manuscript preparation. AJ: contributed to conception and design, carried out manuscript preparation and manuscript review. AS: contributed to conception and design, carried out manuscript preparation and manuscript review. GJT: contributed to conception and design, carried out manuscript preparation and manuscript review. NK: carried out manuscript review. CH: designed the study, carried out manuscript preparation and manuscript review. All authors read and approved the final manuscript.
